# Effectiveness of Peer-assisted learning in medical education: A meta-analysis study

**DOI:** 10.1371/journal.pone.0329605

**Published:** 2025-09-02

**Authors:** Zhuoyang Li, Quan Wang, Junsong Wu

**Affiliations:** Department of Orthopedics, The First Affiliated Hospital, College of Medicine, Zhejiang University, Hangzhou, China; Ajman University, UNITED ARAB EMIRATES

## Abstract

The objective of this meta-analysis was to compare the effects of peer-assisted learning (PAL) and traditional teaching methods on the theoretical knowledge and practical skill education in clinical medicine by summarizing the relevant randomized controlled trial (RCT) studies reported to date. A comprehensive search was conducted across several medical literature databases, including PubMed, EMBASE, Springer, Ovid, Cochrane Library, China National Knowledge Infrastructure (CNKI), and China Biology Medicine Disc (CBMdisc). All pertinent articles, reviews, and references published from January 1970 to May 2023 were analyzed using Review Manager 5.4 software. A total of 81 RCTs were included in this analysis. The findings indicated that PAL surpassed traditional teaching methods in enhancing the transfer of theoretical knowledge and the acquisition of practical skills, especially in terms of student satisfaction and acceptance. It was also observed that, although PAL displayed advantages in certain areas, its effectiveness might not be substantial in fields demanding high levels of specialized knowledge. Furthermore, the reliability of the findings could be constrained by small sample sizes and inadequate implementation of blinding methods. Consequently, it is concluded that PAL is an effective and promising teaching method. Nonetheless, more rigorous research is required in the future to comprehensively explore the role of PAL in medical education.

## 1. Introduction

As the global population expands and ages, there is a notable rise in complex diseases, necessitating elevated standards in medical education. Consequently, clinical medical education has emerged as a critical concern [1,2]. Ensuring the quality of medical professional education and enhancing the practical capabilities of clinical medical personnel present challenging issues that warrant attention in this domain [3]. Traditional pedagogical methods are no longer adequate to address emerging medical needs, prompting leading medical colleges and universities to initiate exploratory teaching reforms. These institutions are striving to employ novel teaching methodologies and establish more comprehensive teaching models with the aim of improving teaching quality. Novel student-centered teaching concepts, including Problem-Based Learning (PBL), Case-Based Learning (CBL), and flipped classrooms, have thus arisen [4,5].

Peer-assisted learning (PAL), a venerable pedagogical approach with origins tracing back to ancient scholars like Socrates and Plato, who utilized mutual questioning and dialogue, is widely implemented today. PAL facilitates a dynamic where students assume the roles of both teachers and learners, thereby enhancing interactive learning [6,7]. Over the past decade, there has been a surge in formal PAL research interest both domestically and internationally, with the merits of PAL increasingly reflected in the growing body of published literature [8]. PAL aids students in better comprehending and applying knowledge. Studies have demonstrated that PAL can significantly enhance students’ academic performance and communication skills [9]. Furthermore, PAL serves to reduce the teaching burden on educators and represents an effective measure for resource conservation. Additionally, PAL boosts students’ confidence and initiative, fosters leadership and teamwork skills [10], and ameliorates the educational environment by encouraging cooperation and communication among students [11].

Although there is a substantial body of literature describing PAL, there is a paucity of objective evaluations of PAL within clinical medical education. Prior studies have yielded some reliable findings, particularly in the context of PAL in medical student education and nursing disciplines [12–14]. Nonetheless, potential biases in study design have not been sufficiently addressed. To the best of our knowledge, this article presents the most extensive meta-analysis of randomized controlled trial (RCT) sample sizes related to PAL in clinical medical education to date.

The purpose of this meta-analysis was to compare the effects and roles of PAL and traditional teaching methods in clinical medical education, encompassing both theoretical knowledge and practical skills, through summarizing the RCT studies of PAL reported to date. It was hypothesized that PAL would yield more favorable outcomes and enjoy greater acceptance compared to traditional teaching methods.

## 2. Methods

### 2.1. Search strategy

Following the Prima Guidelines recommended by the Cochrane Collaboration, this study searched high-quality foreign medical databases, including PubMed, EMBASE, Springer, Ovid, and the Cochrane Library, as well as Chinese medical databases, such as China National Knowledge Infrastructure (CNKI) and China Biology Medicine disc (CBMdisc). All relevant articles, reviews, and reference lists published between January 1970 and May 2023 were searched. Using the PICOS principle and combining keywords, including “medical education,” “peer-assisted learning,” “student tutor,” “clinical medicine,” etc., Boolean operators were also applied to ensure a comprehensive search.

### 2.2. Inclusion and exclusion criteria

The inclusion criteria are as follows:

(1) Study population includes doctors, nurses, or students majoring in clinical medicine or nursing;
(2) Only RCTs were included;
(3) Intervention measures: the control group adopts traditional teaching methods, and the experimental group adopts PAL or teaching methods that include PAL;
(4) Outcome measures: teaching results were evaluated using objective assessment indicators such as theoretical exam scores, skill assessment scores, OSCE or satisfactory scores.

The exclusion criteria are as follows:

(1) Studies that did not adopt the aforementioned intervention measures;
(2) Non-RCT studies;
(3) Duplicate publications, abstracts, lectures, reviews, and case reports were also excluded.

### 2.3. Data extraction and quality assessment

We extracted information including publication date, first author, study design, subject information, research content, evaluation indicators, and other relevant data. RevMan software was used to evaluate the quality of the included studies. Parameters include sequence generation (selection bias), allocation concealment (selection bias), blinding (performance bias), incomplete outcome data (detection bias), selective outcome reporting (reporting bias), and “other issues”. Each item can be classified as “low risk,” “high risk,” or “unclear.” Two reviewers independently assessed the quality of these studies. Any disagreements were resolved by a third reviewer.

### 2.4. Statistical analysis

Statistical analysis was performed using Review Manager 5.4 software (Cochrane Collaboration, Nordic Cochrane Centre, Copenhagen, Denmark). Standardized mean difference (SMD) was used for continuous variables (as different evaluation methods were used between studies). P < 0.05 was considered statistically significant. Homogeneity was tested by Q statistic (significance level at P < 0.1) and I^2^ statistic (significance level at I^2^ > 50%). When there was no significant statistical heterogeneity, a fixed-effects model was used; otherwise, a random-effects model was used. In addition, subgroup analyses were performed on nursing majors, life support training, ultrasound training, surgical skills training, etc.

## 3. Results

### 3.1. Search results

**Fig 1:**
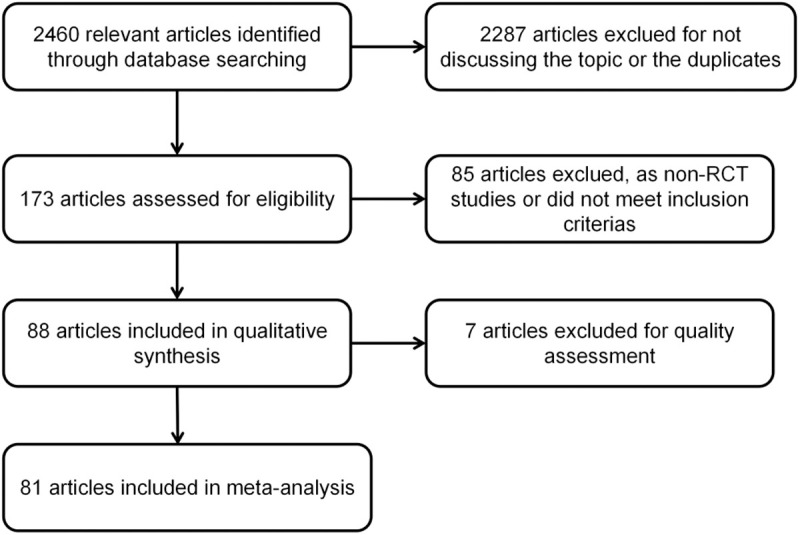
Search strategy flow diagram.

A total of 2460 articles were identified from the database search that met the study objectives. After manual screening and removing duplicate articles, 173 articles were selected. Based on the inclusion and exclusion criteria, 81 studies were selected after reading the full text. These studies were published between 1997 and 2022, with a total of 6752 subjects, including 3412 in the experimental group and 3340 in the control group, with sample sizes ranging from 18 to 356. The literature search process was shown in Fig 1. The basic characteristics of these studies were presented in Table 1. These studies were conducted in various regions globally, including Asia, Europe, and the United States. Most studies were conducted in medical schools and clinical environments, with the majority involving clinical specialties and seven studies involving nursing majors.

**Table 1:** Characteristics of articles included.

References	Subject	Training Context	Tutor Characteristics		Course Characteristics	Duration	Outcomes
			Intervention Group	Control Group			Control Group
Steven 1998 [15]	Medical Student	Physical Diagnosis Course	Fourth-Year Medical Students, GPA ≥ 3.4	Faculty Members	Ten 2-Hour Sessions		Written Test and Skill Examination; Five-Point Likert Scale
Perkin 2002 [16]	Medical And Nursing Student	Basic Life Support	Second-Year Student Instructors	Experienced Clinical Staff	8-hour Course For Four Consecutive Weeks		Multiple Choice Questions (MCQs) For Theoretical Knowledge; Skill Examination
Raupach 2010 [17]	Medical And Nursing Student	ECG Interpretation Skills	Students 1 Year Ahead Of The Learners	Expert Electrocardiographer	6-week Interdisciplinary Cardiorespiratory Module		ECG Assessments
Celebi 2010 [18]	Medical Student	Basic Sonographic Anatomy	Student Tutors	Faculty Staff Sonographer	A 2-Hour Propaedeutic Seminar And A 3-Week Full-Time Clerkship In Ultrasound		Recognized Anatomical Structures By Ultrasound
Dikmen 2017 [19]	Nursing Student	Practical Training	Senior Students	Academic Consultants And Tutors	Skills Training		Objective Structured Practical Ex Amination (OSPE)
Ben-Sasson 2019 [20]	Medical Student	Cardiac Ultrasound Image Acquisition	Teaching Assistants:Third Year Of Clinical Education	A Cardiologist Or A Diagnostic Medical	8-Hour Echocardiography Course	Two Weeks	Practical Exam
Eimer 2020 [21]	Medical Student	Ultrasound Education	The Student Peer-Teacher With Media Support	Ultrasound-Experienced Clinical Teacher Without Media Support	Two Ultrasound Sessions Per Course Scheduled For Two Hours Each		OSCE (Objective Structured Clinical Examination)
Fard 2020 [22]	Nursing Student	Wound Dressing Clinical Skills	Senior Nursing Students With GPA > 17 And Excellent Clinical Skills	Faculty Qualified And Experienced Educators	Seven 5-Hour Sessions	Four Weeks	Researcher-Made Checklist
Kwecharoen 2020 [23]	Medical Student	Electrocardiography Interpretation	The Fourth And Fifth Year Medical Students	Volunteer Residents Or Attending Physicians	One-Hour Study Sessions Weekly	Five Weeks	MCQs
Surabenjawong 2020 [24]	Nursing Student	Airway Management Skill Training	Peer-To-Peer Instructor	Four Experienced Critical Care Nurses	2-Hour Learning Sessions		MCQs; Likert Scale
Kusnoor 2022 [25]	Medical Students	Composing The History Of Present Illness	Resident Facilitators	Faculty Facilitators	The HPI Work Shop		OSCE
Bosse 2010 [26]	Medical Students	Communication Training	Peers	Standardized Patient	Three Training Sessions	Three Weeks	Likert Scale
Schneider 2021 [27]	Medical Students	Communication Training	Peers	Standardized Patient	Two 4-Hour On-Site Sessions	Two Weeks	Likert Scale
Davies 2015 [28]	Medical Students	Electrocardiogram Education	Two Academic Foundation Doctors In Their Second Postgraduate Year	Electronic Learning	Two 30-Minute Sessions		MCQs
Pintér 2021 [29]	Medical Students	Basic Surgical Skills	Sixth-Year Medical Students	Practicing Clinical Physicians	20 × 45 Min Course		Objective Structured Assessment Of Technical Skill (OSATS)
Krause 2017 [30]	Undergraduate Dental Students	Communication Basics Training	Peers	Experienced Psychologist	Three Medical-Dental Interviews		Global Rating Form
Binkhorst 2020 [31]	Medical Students	Pediatric Resuscitation Training	Fifth Or Sixth-Year Medical Students	Proficient And Experienced Pediatricians	Monthly PBLS Training Sessions	Seven Months	VAS; OSCE
Palter 2016 [32]	Medical Students	Laparoscopic Skill	A Faculty Member	Instructional Video+A Laparoscopic Box Trainer	Peer Coaching Before And After Training	Two Weeks	OSATS
Bisesma 2019 [33]	Medical Students	Improve Medical Student's Contributions To Team-Based Projects	Peers	No Peers’ Assessment	Population And International Health (PIH) Team-Based Project		Comprehensive Assessment Team Member Effectiveness (CATME); Likert Short Version Tool
Sevenhuysen 2014 [34]	Physiotherapy Students	Physiotherapy Clinical Education	Student Pairs	Clinical Supervision	Four 2-Hour Workshops	Five Weeks	OSCE; Likert Scale
Divya 2021 [35]	Medical Students	Academic Performance Of The Students	A Previously Trained Final MBBS Students	Tutor	One Hour Session		OSPE
Usman 2019 [36]	Medical Students	Small Group Discussion	10 Willing Students With Academic Scores >75%	Expert Facilitators	Six 40-Minute Topics	From 8am To 1 pm	MCQs
GonzÁLez 2019 [37]	Medical Students	Basic Suture Techniques	Medical Students Previously Trained	Subspecialist Surgeons	Sixteen Academic Hours Divided Into Four Face-To-Face Sessions		OSATS
Ong 2020 [38]	Medical Students	Basic Surgical Skills	Fourth-Year Students	Faculty Members	Two 2-Hour Sessions		Direct Observation Of Procedural Skills (DOPS); Likert Scale
Stephan 2018 [39]	Medical Students	Paediatric Basic Life Support	An Experienced Student Peer Teacher	Video PBLS Lesson	Weekly Training	Nine Weeks	OSCE
Dietsch 2017 [40]	Medical Students	Basic Echocardiography	Peer Medical Students	Video	Two 90-Min Long Sessions		MCQs; OSPE
Nestel 2003 [41]	Medical Students	Patient-Centred Interviewing Skills	Third-Year Students	Medical Teachers	The Simulated Patient Sessions		MCQs
Lemke 2019 [42]	Medical Students	Suturing Skills	2nd Year, Pre-Clerkship Medical Students	Faculty Surgeons	20-Min Instructional Presentation		Time To Profciency (Minutes)
Blank 2013 [43]	Medical Students	Physical Examination	Fourth-Or Fifth-Year Students	Experienced Physician	Eleven 45-Minute Lectures And A 90-Minute Tutorial		OSCE
Tolsgaard 2007 [44]	Medical Students	Clinical Skills	Six Student Teachers From The Skills Centre	Six Associate Professors	Two Hours Of Teaching		OSCE
Haist 1997 [45]	Medical Students	Physical Examination	Fourth-Year Medical Students	Faculty	Ten 2-Hour Sessions		MCQs; Skill Examination; Likert Scale
Widyahening 2019 [46]	Medical Students	Critical Appraisal Skills Learning	Newly Graduated Doctors	Staff Tutors	Lectures, Tutored Group Discussions, And Moderated Plenary Presentations	Four Weeks	MCQs; Evidence-Based Practice Confidence Scale (EPIC)
Shah 2017 [47]	Medical Students	PHYSICAL EXAMINATION	Peer Tutors	Expert	One Hour Session	Four Weeks	OSCE
Rogers 2000 [48]	Medical Students	Surgical Skill Training	Computer Assisted Peer Teaching	Computer-Assisted Learning	A 45-Min Educational Session		Skill Examination
Nomura2017 [49]	Medical Students	Communication Skills	Six Student Tutors From Year 5	Six Physicians	The 10.5-Hour Medical Interview Training Module		OSCE
Kühl 2012 [50]	Medical Students	Focused Emergency Echocardiography	Peer Tutors	Expert Cardiographer	A 12-Hour Basic Echocardiography Course		OSCE
Kassab 2005 [51]	Medical Students	Problem-Based Learning	Peer Tutors	Faculty Tutor	Two Tutorial Sessions	Five Weeks	MCQs; OSPE; A Global Rating Score; Likert Scale
Büscher2013 [52]	Medical Students	Paediatric Examination Techniques	Peer Tutors	Paediatric Tutor	One Hour Session		OSCE
Hudson2008 [53]	Medical Students	Clinical Skills Education	Volunteer Year 6 Students	Paid Doctors	Fourteen Small-Group Structured Tutorials		OSCE
Heckmann 2008 [54]	Medical Students	Neurological Practical Training	Peer Tutors	Postgraduate Tutors	Fifive 30-Min Seminars	One Week	MCQs; OSCE; Likert Scale
Weyrich2009 [55]	Medical Students	Skills Laboratory Training	Senior Student-Tutors	Experienced Faculty Staff	Two 3-Hour Sessions	Two Weeks	OSCE
Knobe 2010 [56]	Medical Students	Musculoskeletal Ultrasound	Nine Sts From Years 3 And 4	Three Ultrasound-Experienced Doctors.	Two Separate 120-Min Lessons		MCQs; OSCE; Likert Scale
Knobe 2012 [57]	Medical Students	Complex Manipulative Motor Skills	Peer Tutors	Two Experienced Physicians	Eight 120-Min Separate Lessons	Eight Weeks	MCQs; OSCE; Likert Scale
Charlier2016 [58]	Master Students	CARDIOPULMONARY RESUSCITATION	Student Pairs	5 Years Of Experience As A Certifified BLS Instructor	54-Min Session		DOPS
Cameron 2015 [59]	Dental Surgery Students	Dental Clinical Skills	Peer Tutors	Experienced Tutor	Two Tasks		OSCE
Vaughn2016 [60]	Surgical Interns	Basic Surgical Skills	Peer Tutors	Faculty	Six 3-Hour In-Person Teaching Sessions.	Twelve Weeks	Global Rating Score
Seifert 2015 [61]	Medical Students	Student-Run Free Clinic Project	Medical Students In Their Final Year Of Clinical Training	Experienced Faculty	Eight 2-H Sessions		OSCE
Pelloux2017 [62]	Medical Students	Peripheral Venous Catheter Insertion Simulation Training	Two Student Tutors	An Anaesthetist Instructor	A 150-Min Session		Student Self-Evaluated Confidence Levels
Cremerius 2019 [63]	Medical Students	Musculoskeletal Ultrasound Skills	Peer Tutors	A DEGUM-Certifed Doctor With Several Years Of Ultrasound Experience	Two 120-Min Long Sessions		MCQs; OSCE
Kronschnabl 2021 [64]	Medical Students	Cardiovascular Physical Examination	A 4th-Year Medical Student	Faculty	Five 90-Min Sessions And 75 -Min Simulator Training		A Standardized Checklist
Alsulmi 2022 [65]	Medical Students	Virtual Chest X-Ray Interpretation	An Experienced Sixth-Year Medical Student	A Radiology Faculty Member	Two 2-hour Sessions		MCQs
Rui 2021 [66]	Medical Students	The Clinical Teaching Of Vertigo/Dizziness-Related Diseases	Student Tutors	Seven Attending Doctors And 6 Senior Doctors		Two Weeks	MCQs
Ting 2018 [67]	Medical Students	International Student Clinical Practice In Obstetrics And Gynecology	Second-Year Graduate Student	Secondary Physician	Clinical Practice		Written Test And Skill Examination
Bin 2022 [68]	Residents	Standardized Training For Intensive Care Medicine Residents	Student Pairs	The Intensive Care Medicine Doctors With More Than 3 Years Of Attending Experience	Teaching Course Training	Two Days	Written Test And Skill Examination
Yue 2019 [69]	Medical Students	Basic Medical Experimental Cardiovascular Teaching	Study Group	Faculty	Five 200-Min Teaching Modules		Written Test And Skill Examination
Feng 2021 [70]	Residents	Cultivation Of Critical Thinking Skills	Study Group	Faculty	4-hour Training + 8 PAL Sessions	Two Months	Min-Cex; Critical Thinking Disposition Inventory-Chinese Version (CTDI-CV)
Ping 2017 [71]	Medical Students	Clinical Practice In Neurology	Study Group	Faculty With More Than 3 Years Of Attending Experience	2-week Clinical Practice		MCQs; Skill Examination
Yuan 2021 [72]	Medical Students	Clinical Skills Teaching Of Trainee Doctors	Study Group	Faculty	8-hour Teaching		Skill Examination
Ying 2021 [73]	Medical Students	Otolaryngology Teaching For International Students	Study Group	Faculty	18-hour Theoretical Course + 12-hour Practice Course		MCQs; Skill Examination; Likert Scale
Pin 2021 [74]	Nursing Student	Infectious Disease Teaching	Study Group	Faculty	Theory Combined With Operation Teaching Once A Week	One Month	360°Checklist
Qun 2021 [75]	Medical Students	Neurosurgery Teaching	Study Group	Faculty	10-hour Theoretical Course + 8-hour Practice Course		MCQs; Skill Examination
Ning 2020 [76]	Residents	Standardized Training Of Emergency Medicine	Study Group	Faculty			MCQs; Skill Examination
Meng 2019 [77]	Nursing Student	Basic Nursing Teaching	Outstanding Students Serve As Group Leaders And Peer Tutors	Faculty			MCQs; Skill Examination
Ke 2022 [78]	Medical Students	Clinical Internship In Neurology	Study Group	Teacher And MOOC Platform	Clinical Practice	Two Weeks	MCQs; Skill Examination
Li 2020 [79]	Medical Students	Teaching Of Obstetric Physical Examination	Fifth-Year Medical Students In Clinical Internship	Senior Attending Doctors In Obstetrics And Gynecology	Theory Teaching + Skill Demonstration		OSCE
Zhao-1 2019 [80]	Medical Students	Cultivation Of Surgical Clinical Thinking Ability	Peer Tutors	Faculty	Skill Demonstration		CTDI-CV
Zhao-2 2019 [81]	Medical Students	Practical Teaching Of Surgery	Peer Tutors	Faculty	Skill Demonstration		MCQs; Skill Examination
Wang 2016 [82]	Nursing Student	Emergency Skills Training	Senior Students With Proficient Emergency Operation Skills	Faculty	Six 45-Min Courses		Skill Examination
Gao 2019 [83]	Medical Students	Teaching Of Basic Operations And Aseptic Techniques In Surgery	Student Pairs	Faculty			Skill Examination
Xing 2020 [84]	Nursing Student	Clinical Teaching For Operating Room Nurses	Study Group	Faculty			Nursing Professional Core Competence Scale; Written Test And Skill Examination; Learning Effectiveness
Mi 2017 [85]	Medical Students	Clinical Practicum And Teaching For Critical Care Stroke Patients	Study Group	Specialist Attending With More Than 5 Years Of Clinical Work Experience			MCQs
Ma 2020 [86]	Medical Students	Teaching Of Physical Examination	Student Pairs	Specialist Deputy Director With PAL Teaching Experience	Theory Teaching + Skill Demonstration + Case Analysis		MCQs
Chen 2019 [87]	Medical Students	Teaching Of Lumbar Puncture Skills	Study Group	Faculty With Solid Theoretical Knowledge And Rich Teaching Experiences	2-hour Course		Written Test And Skill Examination
Li-1 2018 [88]	Medical Students	Teaching Of Midwifery Skills Training	Study Group	Attending Or Chief Nurse			Written Test And Skill Examination
Zhang-1 2019 [89]	Residents	Standardized Training For Endocrinology Resident Physicians	Study Group	Attending Or Above	8-week Course		Written Test And Skill Examination
Dong 2020 [90]	Medical Students	Internal Medicine Clinical Internship For International Students	Study Group	Faculty			Written Test And Skill Examination
Wang 2021 [91]	Medical Students	Teaching Of Anesthesiology Undergraduate Graduation Internship	Study Group	Senior Attending With More Than 3 Years Of Experience	16-Week Internship Program		Written Test And Skill Examination
Li-2 2018 [92]	Medical And Nursing Student	Self-Learning Ability	2014th Master’s Degree Graduate Student	Faculty	Six 1.5/2-hour Sessions		Likert Scale
Zhang 2018 [93]	Medical Students	Clinical Internship And Teaching In Urology Surgery	Master’s Degree Graduate Student At The Same Time	Faculty			Written Test And Skill Examination
Zhang-2 2019 [94]	Medical Students	Clinical Internship In Surgery	10 Outstanding Interns With Excellent Theoretical And Skill Results	Faculty			MCQs
Zhang 2021 [95]	Medical Students	Emergency Rescue Training	Peer Tutors	Faculty			Written Test And Skill Examination

### 3.2. Quality assessment of included studies

The quality of the included studies was evaluated. As shown in Table 1, bias assessment (according to the Cochrane Handbook for Systematic Reviews of Interventions 5.0) was conducted for the 81 RCT studies included. The entire evaluation was performed by two reviewers independently, and any disagreements were resolved by a third reviewer.

**Fig 2:**
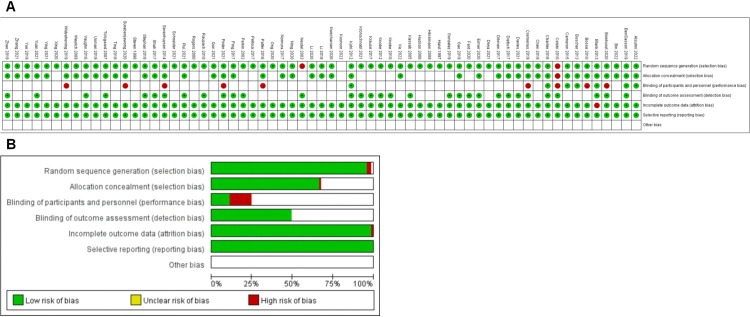
Results of bias in the included studies.

Although the included studies had high quality, due to the nature of study design and intervention measures, participants could not be well blinded in all studies, indicating concerns with regards to participant blinding bias in most literature, with 11 articles exhibiting high risk, as shown in Fig 2.

### 3.3. Meta-analysis results

#### 3.3.1. Theoretical knowledge.

**Fig 3:**
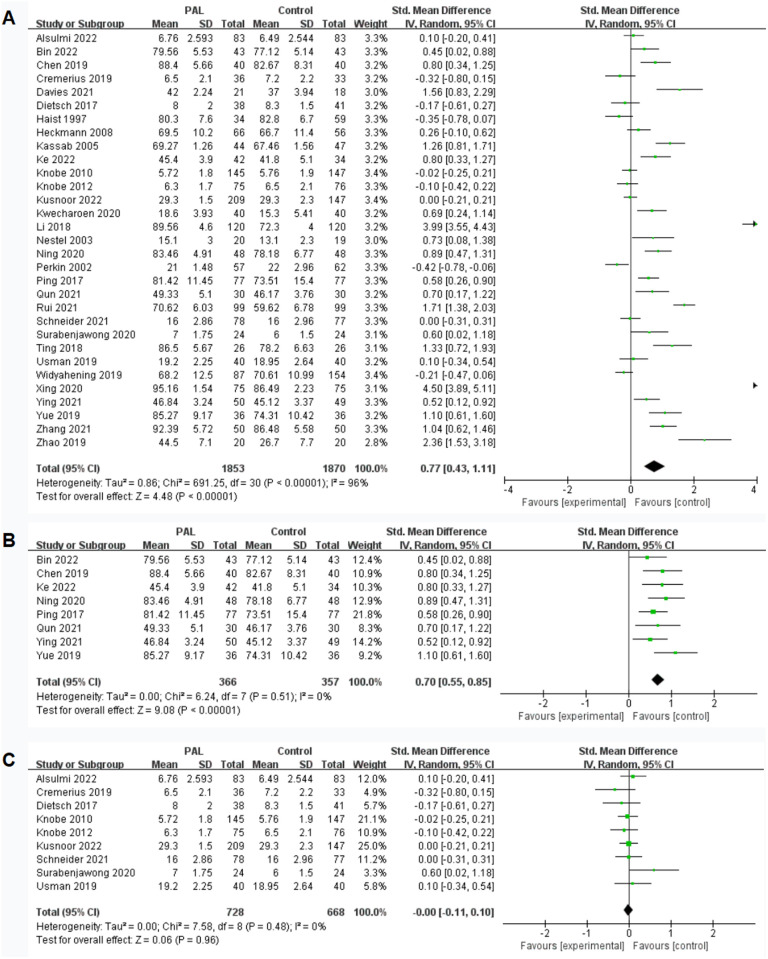
Forest plot for effect of PAL on theoretical knowledge.

A total of 31 studies involved the impact of PAL on theoretical knowledge transfer, with the theoretical knowledge assessment results for the two groups being 7–95.16 points and 6–86.49 points, respectively. The results showed significant differences (SMD = 0.77, 95% CI: 0.43–1.11, P < 0.00001), favoring the PAL group. However, there was considerable heterogeneity between the two groups (I^2^ = 96%), which may be related to the different evaluation methods used in various studies, not all of which used multiple choice questions (MCQs) in the hundred-mark system. The results were shown in Fig 3a.

Considering that heterogeneity may be related to different evaluation methods, we conducted subgroup analysis to determine the source. When we summarized studies using 100-point scales for subgroup analysis, the results showed significant differences (SMD = 0.70, 95% CI: 0.55–0.85, P < 0.00001, I^2^ = 0%), favoring the PAL group. The results were shown in Fig 3b. When we summarized studies using 20-point scales for subgroup analysis, the results showed no significant differences between the two groups (P = 0.96, I^2^ = 0%). The results were shown in Fig 3c.

#### 3.3.2. Procedural skill.

**Fig 4:**
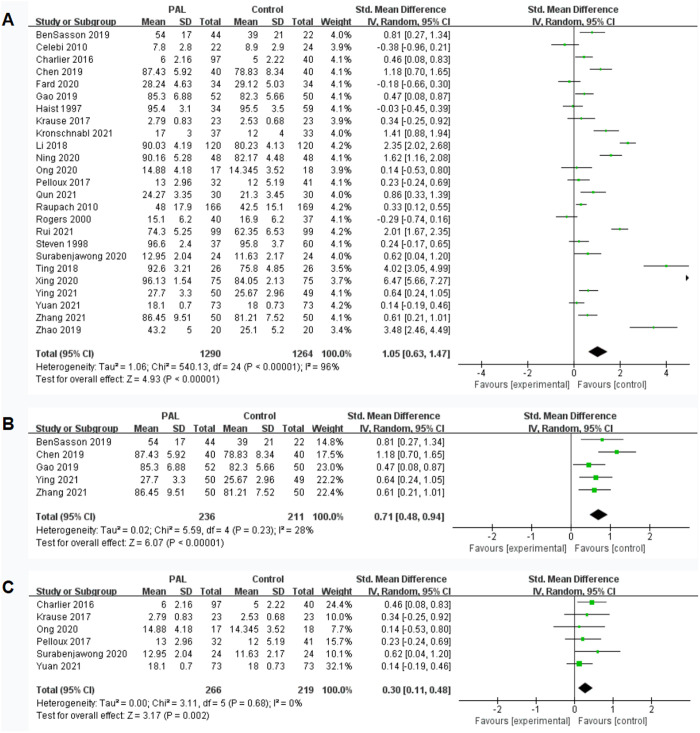
Forest plot for effect of PAL on procedural skill.

A total of 25 studies involved the impact of PAL on skill performance, with the skill assessment results for the two groups being 6–96.13 points and 5–84.05 points, respectively. The results showed significant differences (SMD = 1.05, 95% CI: 0.63–1.47, P < 0.00001), favoring the PAL group. However, there was considerable heterogeneity between the two groups (I^2^ = 96%), which may be related to the different evaluation methods used in various studies. The results were shown in Fig 4a.

Considering that heterogeneity may be related to different evaluation methods, we conducted subgroup analysis to determine the source. When we summarized studies using 100-point scales for subgroup analysis, the results showed significant differences (SMD = 0.71, 95% CI: 0.48–0.94, P < 0.00001, I^2^ = 28%), favoring the PAL group. The results were shown in Fig 4b. When we summarized studies using 20-point scales for subgroup analysis, the results showed significant differences (SMD = 0.30, 95% CI: 0.11–0.48, P = 0.002, I^2^ = 0%), favoring the PAL group. The results were shown in Fig 4c.

#### 3.3.3. OSCE.

**Fig 5:**
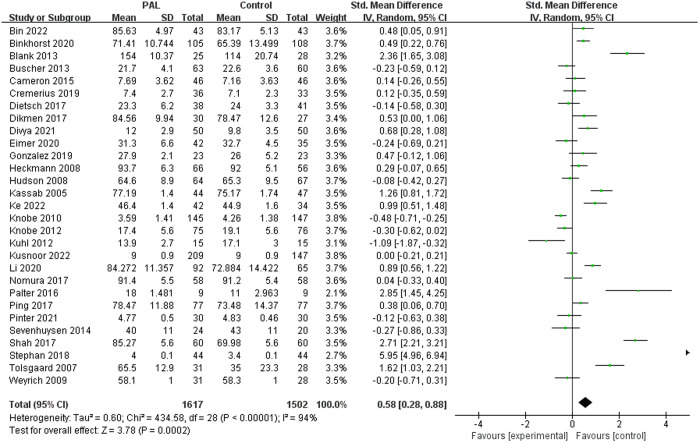
Forest plot for effect of PAL on OSCE.

A total of 29 studies involved the impact of PAL on OSCE, with the OSCE assessment results for the two groups being 4−154 points and 3.4−114 points, respectively. The results showed significant differences (SMD = 0.58, 95% CI: 0.28–0.88, P = 0.0002), favoring the PAL group. However, there was considerable heterogeneity between the two groups (I^2^ = 94%), which may be related to the different OSCE station designs and evaluation criteria used in various studies. The results were shown in Fig 5a.

Considering that heterogeneity may be related to different evaluation methods, we conducted subgroup analysis to determine the source. When we summarized studies using 100-point scales for subgroup analysis, the results showed significant differences (SMD = 0.37, 95% CI: 0.23–0.51, P < 0.00001, I^2^ = 0%), favoring the PAL group. The results were shown in Fig 5b. When we summarized studies using 20-point scales for subgroup analysis, the results showed no significant differences between the two groups (P = 0.92, I^2^ = 0%). The results were shown in Fig 5c.

#### 3.3.4. Satisfaction.

**Fig 6:**
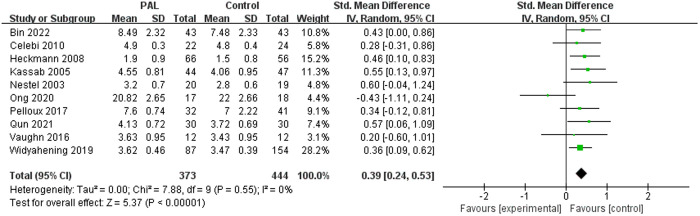
Forest plot of satisfaction on different teaching methods.

A total of 10 studies involved the evaluation of satisfaction of PAL, with the satisfaction assessment results for the two groups being 1.9–20.82 points and 1.5–7.48 points, respectively. The results showed significant differences (SMD = 0.39, 95% CI: 0.24–0.53, P < 0.00001, I^2^ = 0%), favoring the PAL group, which means in most studies, subjects were more satisfied with PAL than with traditional teaching methods. The results were shown in Fig 6.

#### 3.3.5. Basic life support.

**Fig 7:**
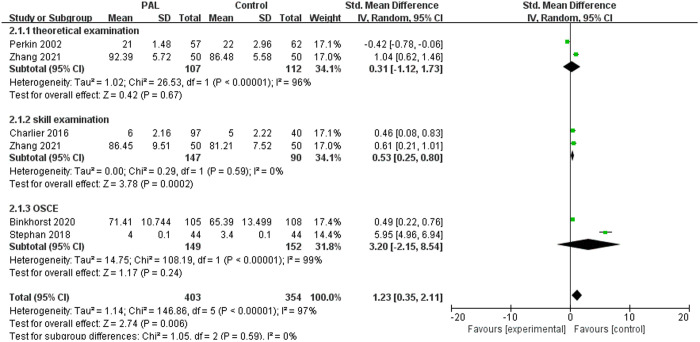
Forest plot for the effects of PAL on basic life support.

We further conducted subgroup analysis based on the content of the studies. There were 5 articles involving basic life support training, such as CPR and pediatric life support. The meta-analysis results showed that PAL had a significantly better teaching effect on basic life support than the control group (SMD = 1.23, 95% CI: 0.35–2.11, P = 0.006), with considerable heterogeneity (I^2^ = 97%). Considering that heterogeneity may be related to different evaluation methods, we conducted subgroup analysis to determine the source. The results showed that PAL did not show significant differences for theoretical knowledge teaching and OSCE, although the results appeared to favor the PAL group. However, for skill performance on basic life support, the PAL group had a significant advantage (SMD = 0.53, 95% CI: 0.25–0.80, P = 0.0002), with excellent homogeneity (I^2^ = 0%). The results were shown in Fig 7.

#### 3.3.6. Ultrasound teaching.

**Fig 8:**
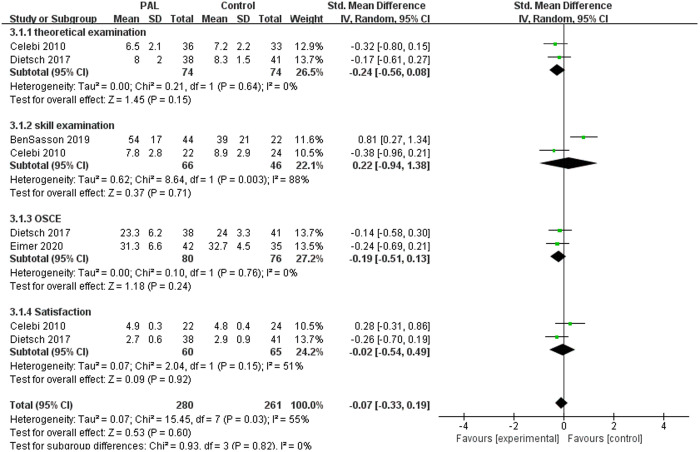
Forest plot for the effects of PAL on ultrasound teaching.

There were 4 studies involving ultrasound teaching, such as cardiac ultrasonography and musculoskeletal ultrasonography. The meta-analysis results showed no significant difference between the two groups (SMD = −0.07, 95% CI: −0.33–0.19, P = 0.60, I^2^ = 55%). We further conducted subgroup analysis of these 4 studies. The results showed that there was no significant difference between the two groups in theoretical knowledge, skill performance, OSCE and satisfaction (P > 0.05). From the perspective of ultrasound teaching, PAL did not show superiority over traditional teaching methods. The results were shown in Fig 8.

#### 3.3.7. Surgical skills.

**Fig 9:**
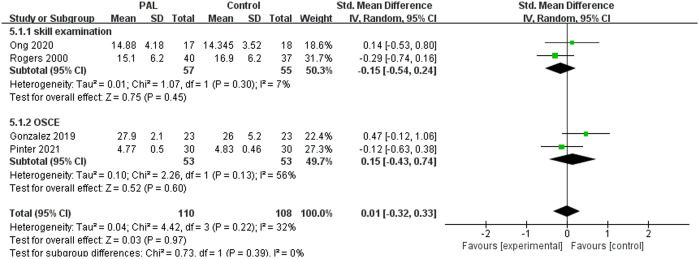
Forest plot for the effects of PAL on surgical skills.

There were 4 studies involving surgical skills teaching, such as suturing, sterile techniques, and laparoscopic techniques. The meta-analysis results showed no significant difference between the two groups (SMD = 0.01, 95% CI: −0.32–0.33, P = 0.97, I^2^ = 32%). Further subgroup analysis of these 4 studies showed that there was no significant difference between the two groups in terms of skill performance and OSCE (P > 0.05). From the perspective of surgical skills, PAL did not show superiority over traditional teaching methods. The results were shown in Fig 9.

#### 3.3.8. Clinical data collection.

There were 7 studies involving clinical data collection, such as medical history taking and physical examination. The meta-analysis results showed no significant difference between the two groups (SMD = −0.11, 95% CI: −0.25–0.02, P = 0.10, I^2^ = 38%).

**Fig 10:**
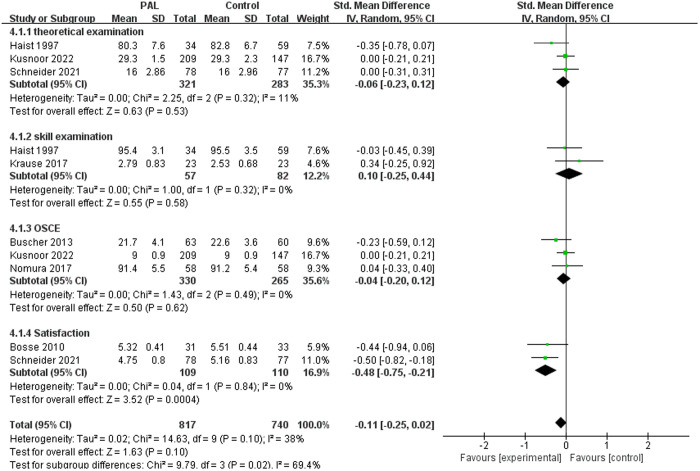
Forest plot for the effects of PAL on clinical data collection.

Further subgroup analysis of these 7 studies showed that there was no significant difference between the two groups in terms of theoretical knowledge, skill performance, and OSCE (P > 0.05, I^2^ = 0–11%). From the perspective of clinical data collection, PAL did not show superiority over traditional teaching methods.The results were shown in Fig 10.

#### 3.3.9. Nursing education.

**Fig 11:**
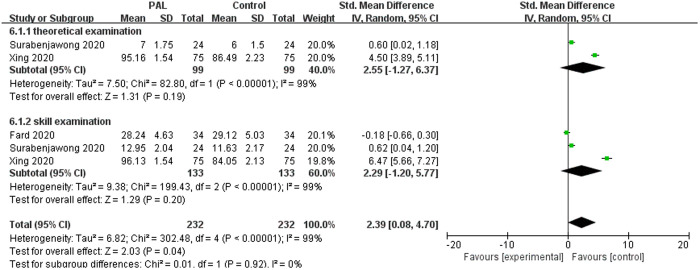
Forest plot for the effects of PAL on nursing education.

Three studies on nursing education were included in the meta-analysis, and the results showed that PAL had a significantly better teaching effect on nursing education than the control group (SMD = 2.39, 95% CI: 0.08–4.7, P = 0.04). Although subgroup analysis showed no significant difference between the two groups in terms of theoretical knowledge and skill performance (P > 0.05). The results were shown in Fig 11.

## 4. Discussion

This review meticulously analysis the impact of PAL within the realm of medical education. It compellingly demonstrates that PAL surpasses conventional teaching methodologies in disseminating theoretical knowledge and guiding practical skill acquisition. It is particularly encouraging to observe the marked preference and satisfaction among students for PAL over traditional methods, laying a robust foundation for its broader adoption. Nonetheless, in disciplines requiring advanced and specialized knowledge, the superiority of PAL is not as pronounced. In conclusion, PAL emerges as a commendable and potent educational approach, although further investigation is warranted to fully understand its applicability across the diverse spectrum of medical education.

These findings are consistent with extant literature, underscoring the importance of PAL as an active learning instrument in medical education. PAL strategies can aid students in cultivating critical competencies such as cooperation, critical exploration, and reflection [13]. Durning’s cognitive consistency hypothesis elucidates the efficacy of PAL even among peers with similar educational backgrounds [96]. The comparable baseline knowledge levels among peers may facilitate better understanding than guidance from teachers alone. Varied learning environment conditions can also influence learning effectiveness: within a peer group, students tend to be more open and less defensive, free from the fear of criticism or ridicule [57]. Furthermore, the reciprocal role-switching among peers not only augments their sense of responsibility and initiative but also refines their communication skills and teamwork abilities. Such positive social interactions contribute to enhancing students’ emotional intelligence [97]. Simultaneously, PAL can effectively stimulate students’ critical thinking: students are required not only to analyze and evaluate the viewpoints presented by their peers but also to assimilate information from diverse perspectives, fostering multifaceted thinking. Additionally, students must reflect on and summarize their learning processes and outcomes, which significantly contributes to the development of their critical thinking skills [98].

Furthermore, PAL offers advantages not only to students but also to peer tutors. As Ross famously remarked, “Teaching is learning twice” [99]. There are disparities in the learning processes of teachers and students, and by teaching others, peer tutors can facilitate more detailed and specific learning of course content, leading to long-term knowledge retention [100]. Considering the high memory demands of medical education, participation in PAL can enhance learning and augment clinical experience while teaching, thereby easing the burden of exams. Peer tutors are also required to provide learning materials to other students, which leads them to become familiar with and simplify the learning content [101]. These potential benefits undoubtedly position PAL as a valuable tool in medical education. Cohen et al. discovered that students participating in PAL generally exhibit a higher level of satisfaction with the course, as they experience a greater sense of autonomy and support during the learning process. Students not only acquire knowledge from their peers but also receive emotional support, which is relatively scarce in traditional teaching models [102,103]. This is consistent with our research findings, which indicate that the satisfaction and acceptance of PAL are significantly superior to those of traditional teaching models.

However, some literature also indicates that PAL does not exhibit a significant advantage in knowledge acquisition [45,104]. This phenomenon may be attributed to the limited professional knowledge level of peer tutors. Teaching professional theoretical and clinical scientific knowledge necessitates a higher degree of expertise, which peer tutors might not possess, potentially hindering their ability to provide in-depth explanations and diverse perspectives. Regarding specific professional clinical skills, peer tutors may not demonstrate a superior status compared to their expert peers. On the other hand, studies that show no significant differences in overall analyses often have relatively small sample sizes, which could introduce potential biases. Nevertheless, some research posits that this situation does not necessarily lead to negative outcomes. When peer tutors encounter questions or are unable to answer their peers’ inquiries, they tend to invest additional effort in studying and learning, thereby further enhancing their scores. This hypothesis is referred to as “mindful acceptance” [105]. This phenomenon could explain why, even in the absence of significant differences, the results generally tend to favor PAL.

Interestingly, this review’s subgroup analysis revealed that, in comparison to traditional teaching methodologies, PAL did not markedly enhance students’ pass rates in more specialized skill instruction, such as life support, ultrasound, surgical skills, and the like. This finding aligns with previous literature. Mundell et al. posited that cardiopulmonary resuscitation techniques within life support should be classified as advanced surgical skills, necessitating a series of professional responses and decisions in response to sudden changes in patient physiology [106]. Consequently, Zhang advocated that more intricate clinical skills demand mentors to possess ample professional knowledge and experience to conduct courses effectively, while also providing appropriate assessment of student performance and cognition [1]. Furthermore, the spontaneous assimilation of concepts and content is challenging when learning complex tasks, often necessitating a mentor’s explanation and linkage. This undoubtedly poses a challenge for peer tutors. Most studies outlining pre-service training for peer tutors are confined to limited time and knowledge within a restricted curriculum scope [107]. However, this does not negate the potential of PAL in teaching advanced skills. Peer tutors can still effectively deliver some procedures and content traditionally performed by professional tutors. Alternatively, longer and more comprehensive pre-service training may also positively impact the effects of PAL, although considerations of human and time costs must be taken into account.

In the implementation of teaching activities, “cost” is another crucial consideration. However, only a few studies analyze the cost-effectiveness of PAL. Evaluating educational outcomes and cost – effectiveness is essential to ascertain whether a teaching method is appropriate and reasonable. Economic analysis plays a significant role in decision – making regarding medical resource allocation [108]. Lemke discovered in a study on surgical suture skills training that both PAL and traditional teaching methods achieved good teaching objectives. Nevertheless, PAL can reduce teacher costs by training peer tutors. Moreover, if the “train - the - trainer model” is introduced, that is, training more future peer tutors through peer tutor training, it can further eliminate teacher costs. And it is believed that this makes better use of clinical equipment and reduces maintenance and repair costs [42]. This can be regarded as another advantage of PAL.

Compared to conventional teaching methodologies, PAL also exhibits its own set of limitations. The efficacy of PAL is, to a certain degree, contingent upon the ability levels of the peers involved. In instances where there is a substantial disparity in abilities among peers, it may prove to be difficult for students of lower abilities to derive benefits from the guidance of their higher-achieving peers. Furthermore, given that teachers typically assume the role of facilitators, the absence of systematic and structured guidance during the initial phases of PAL may leave some students uncertain about the trajectory of their learning. Additionally, owing to the diverse and interactive nature of the learning process, traditional testing and evaluation methodologies may not comprehensively capture the learning outcomes of students engaged in PAL [98]. This could potentially explain the considerable heterogeneity observed in some of the results of this study.

To further optimize the efficacy of PAL, several recommendations can be made. Firstly, it is advisable to offer training to peer tutors on essential skills, including communication, feedback techniques, and instructional guidance, prior to PAL sessions. Secondly, PAL can be integrated with conventional teaching methodologies, thus combining the professionalism and systematic approach of traditional teaching with the autonomy and adaptability inherent in PAL. Furthermore, a comprehensive evaluation of PAL should be implemented, utilizing multiple assessment methods such as self-assessment, peer assessment, and teacher assessment at the conclusion of the teaching period, as well as by analyzing students’ engagement and the quality of interactions throughout the instructional process. It is posited that PAL, as an innovative educational paradigm, is poised to emerge as a pivotal teaching strategy in future educational endeavors, thereby affording students enriched and diversified learning experiences.

## 5. Limitations

This review exhibits several limitations. Owing to the extensive volume of literature and the diversity of evaluation methodologies employed, the heterogeneity within the meta-analysis remains considerably high, despite endeavors to mitigate this through the selection of RCTs and subgroup analyses. Moreover, there are certain unavoidable biases, encompassing participant bias and observer bias. The behavior and reactions of participants may be swayed by expectancy effects, suggesting that the study outcomes might reflect participants’ anticipated responses rather than the actual effects of the educational interventions themselves. Furthermore, when observers are cognizant of group assignments, their recording and analytical processes may exhibit a tendency to support the hypothesis, potentially diminishing the accuracy and reliability of the data. Another limitation pertains to the absence of a standardized research design and the inability to regulate the training and quality of peer mentors. Lastly, the review solely assessed the role of PAL in medical education, without exploring its impact in other professions or disciplines. In light of these limitations, it is not possible to conclusively ascertain through simple quantitative analysis whether PAL is superior or inferior to traditional teaching methods. Instead, we can only qualitatively propose that the effectiveness of PAL is not inferior to that of traditional teaching methods and may potentially offer advantages in certain aspects.

## 6. Conclusion

This review provides a comprehensive summary of the efficacy of PAL within medical education, affirming that PAL surpasses conventional teaching methodologies in the realms of theoretical knowledge dissemination and procedural skill mentorship. Evidently, PAL emerges as an effective and promising educational approach. Nonetheless, further rigorous investigations are warranted in the future to comprehensively elucidate the role of PAL in medical education.

## Supporting information

S1 File PRISMA_2020_checklist. (DOCX)

S2 File Characteristics of included studies and risk of bias. (XLSX)

S3 File Study selection table. (XLSX)
